# Quality of care and job satisfaction in a Hospital Trust before and after The Coordination Reform in Norway

**DOI:** 10.1002/nop2.554

**Published:** 2020-07-05

**Authors:** Irene Aasen Andersen, Ole T. Kleiven, Lars Kyte, Marny Alice Solhaug Pettersen

**Affiliations:** ^1^ Faculty of Health Studies Western Norway University of Applied Sciences Førde Norway; ^2^ Department of Medicine Førde Central Hospital Førde Norway

**Keywords:** job satisfaction, nurse, quality of patient care, specialist health service, The Coordination Reform

## Abstract

**Aims:**

To study the impact of organizational changes on the quality of health services and on health professionals' job satisfaction in specialist health services.

**Design:**

A repeated cross‐sectional study, including 5 years before (2007) and 5 years after (2017) the introduction of The Coordination Reform in Norway.

**Methods:**

Nurses and auxiliary nurses working in medical wards at three hospitals evaluated the quality of health services and various aspects of their working conditions, using questionnaires: Quality of Patient Care and the Job Satisfaction Scale.

**Results:**

In 2017, nurses and auxiliary nurses had longer work experience compared with 2007. Nurses and auxiliary nurses also worked full hours. There was no significant change over time in total Quality of Patient Care score or in any of the sub‐scores. There was no significant change in total Job Satisfaction Scale score over time, but there was a significant decline in the sub‐score for physical working conditions.

## 1 INTRODUCTION

Norwegian health care is under pressure due to extensive changes in the organization and distribution of tasks between primary and specialized health services (Helsedirektoratet, 2012; Skogli, Theie, Nellemann, Jakobsen, & Flateland, 2017). Since the introduction of The Coordination Reform in Norway in 2012, the use of resources in specialized Health service has been streamlined and more tasks have been transferred to municipal health and care services (Norwegian Ministry of Health & Care Services, 2008–2009). Patients have fewer hospital stays than before and many patients now receive treatment and follow‐up in primary care that they had received in the hospital (Tyrholm, Kvangarsnes, & Bergem, 2016).

In the years leading up to 2030, an ever‐increasing deficit in health personnel is expected (Helsedirektoratet, 2012) at the same time the number of elderly people is expected to substantially increase (Helsedirektoratet, 2012). Experience shows that patients over 70 years of age use hospital services five times more than rest of the population (Førde, 2018). It is also said that the need for health personnel in the specialized Health services will decrease (Helsedirektoratet, 2012). A study by Aiken et al. (2014) of nurses at 300 hospitals in nine countries found that increased workload increased the odds of patient mortality. Given this finding, it is important to pay attention to how health personnel in hospitals assess the quality of care and their job satisfaction in a healthcare system that is constantly changing.

The professionals that provide care to patients in hospital wards are primarily nurses and auxiliary nurses (Helsedirektoratet, 2012). Nurses are educated in a university or university college (Kunnskapsdepartementet, 2008), and auxiliary nurses have a vocational education (Utdanningsdirektoratet, 2019).

## 2 BACKGROUND

The competence of nurses and the quality of patient care are closely correlated and linked to the values of one's professional identity (Christiansen & Bjørk, 2016; Kleiven, Kyte, & Kvigne, 2016). Nurses' own evaluations of care quality are associated with the 30‐day survival of patients after hospital admission. This indicates that the microsystem nurses are a part of can mirror the overall performance of a hospital (Tvedt, Sjetne, Helgeland, & Bukholm, 2014).

Quality of care has been found to be related to health, environment and safety in an organization (Helse‐ og omsorgsdepartementet, 2018–2019; Tvedt et al., 2014), and good nursing is related to organizational factors, the work environment and professional obligations (Lu, Barriball, Zhang, & While, 2012; Wan, Li, Zhou, & Shang, 2018). Hospital wards that have high‐quality leadership, which includes interdisciplinary teamwork and staff who are satisfied with their jobs, tend to have lower patient mortality, better patient safety and more satisfied patients (Aiken et al., 2011, 2014; Ball et al., 2018; Mwachofi, Walston, & Al‐Omar, 2011). A good working environment is an important prerequisite for job satisfaction (Copanitsanou, Fotos, & Brokalaki, 2017), and poor well‐being (e.g. burnout) among health professionals leads to reduced quality of care and patient safety (Hall, Johnson, Watt, Tsipa, & O'Connor, 2016). This suggests that the system and routines can influence how one delivers sound and good services. For example, working conditions in hospitals have an impact on patients' risk of adverse events (Sjetne, Veenstra, Ellefsen, & Stavem, 2009).

Norway is well above average in terms of patient coverage by doctors and nurses, and it is the country with the most employees in the health and social sector, as a whole (OECD, 2018). Even though the Norwegian health system has more resources, Norway's quality of care is close to the average of OECD countries. One of the goals of The Coordination Reform was to achieve better health and care services in a sustainable way (Norges forskningsråd, 2016). Since the reform was introduced in 2012, more patients have been admitted to hospitals while the length of stay has decreased (Norges forskningsråd, 2016). The added value per employee has increased without the number of re‐admissions having changed. A further reduction in length of stay is expected in the years leading up to 2025 (Helsedirektoratet, 2012).

There is little information about the quality of patient care since the introduction of The Coordination Reform (Riksrevisjonen, 2016). We do not know of any study that has tracked quality of care and job satisfaction over time in relation to the changes imposed by the reform, so there is reason to believe the present study fills in knowledge gaps in the literature. The results should be of interest to many people who are responsible for health services, clinical practice and education.

### 2.1 Research question

Did nurses and auxiliary nurses working in medical wards rate the quality of health services and their job satisfaction differently 5 years before (2007) and 5 years after (2017) the introduction of The Coordination Reform in Norway?

## 3 THE STUDY

### 3.1 Design

A repeated cross‐sectional study was conducted where data from 2007, 5 years before The Coordination Reform, were compared with data from 2017, 5 years after the reform. The inclusion criteria were nursing staff who were employed on inpatient medical wards of three hospitals owned by the Førde Hospital Trust in Norway.

### 3.2 Methods

All the nursing staff were invited to participate in the study. The 2007 sample and the 2017 sample completed the same self‐administered battery of questionnaires. Descriptive variables of the samples were as follows: which ward they worked on (A, B or C), profession (nurse or auxiliary nurse), total years of work experience (<1 year, 1–3 years, 3–5 years, 5–10 years or ≥10 years), work experience on the ward (<1 year, 1–3 years, 3–5 years, 5–10 years or ≥10 years), continuing education (no or yes) and position size (<50%, 50%–99% or 100%).

The Quality of Patient Care (QPC) questionnaire was used to measure the extent to which nurses thought their patients' basic needs were met by the hospital. The six QPC items are as follows: (1) basic physiologic needs; (2) need for communication and contact with others; (3) need for sleep, rest and peace; (4) need for infection control and hygiene; (5) ensuring correct treatment, care and prevention of adverse events; and (6) counselling on health promotion and adequate use of healthcare facilities. How well the patients' basic needs were met was rated from 1 (very poor)–7 (very good). An average score was calculated based on the six questions (the sum of the six items divided by 6). The QPC was developed by the Norwegian Knowledge Centre for the Health Services and validated in a Norwegian context (Sjetne et al., 2009). In our study, Cronbach's alpha of QPC was 0.83.

The Job Satisfaction Scale (JSS) was used to measure job satisfaction. The 10 JSS items on job satisfaction are as follows: (1) the amount of responsibility given; (2) variation in work; (3) colleagues and fellow workers; (4) physical working conditions; (5) opportunities to use one's skills; (6) overall job situation; (7) freedom to choose one's own methods of working; (8) recognition one get for one's achievements; (9) rate of pay; and (10) work hours. Each item was rated from 1 (very dissatisfied)–7 (very satisfied). An average score was calculated (the sum of the 10 items divided by 10). The JSS is a validated scale for nurses in the Norwegian context (Andersen & Andersen, 2012). In our study, Cronbach's alpha of JSS was 0.86.

### 3.3 Analysis

Categorical variables are presented as frequencies and percentages, and continuous variables are presented as means and standard deviations (SDs). Differences in characteristics between the 2007–2017 samples were analysed with the chi‐square test. Simple and multiple linear regression (adjusting for the variables in Table 1) were used to analyse differences in QPC and JSS scores between the 2007–2017 samples. These results are presented as average differences with 95% confidence intervals. The average scores on the QPC and JSS are the main outcomes of this study, while the results for changes in single‐item questions are secondary outcomes. Adjustments for multiple testing were not made; exact *p*‐values are reported. Statistical analyses were performed with SPSS software for Windows, version 25.

**TABLE 1:** Comparison of the characteristics of the 2007 and 2017 samples

Variables	2007, *N* = 86	2017, *N* = 75	*p*‐value
Participants by ward, *N* (%)			
Ward A	52 (61)	28 (37)	.009
Ward B	13 (15)	23 (31)	
Ward C	21 (24)	24 (32)	
Profession, *N* (%)			
Nurse	58 (67)	57 (76)	.003
Axillary nurse	28 (33)	12 (16)	
Data missing	0 (0)	6 (8)	
Total work experience, *N* (%)			
<1 year	7 (7)	2 (3)	.058
1–3 years	11 (13)	5 (7)	
3–5 years	6 (7)	7 (9)	
5–10 years	16 (19)	6 (8)	
≥10 years	40 (47)	51 (68)	
Data missing	6 (7)	4 (5)	
Work experience in the ward, *N* (%)			
<1 year	11 (13)	4 (5)	.069
1–3 years	11 (13)	10 (13)	
3–5 years	11 (13)	11 (15)	
5–10 years	24 (28)	10 (13)	
≥10 years	27 (31)	38 (51)	
Data missing	2 (2)	2 (3)	
Continuing education, *N* (%)			
No	55 (64)	48 (64)	.930
Yes	24 (28)	22 (29)	
Data missing	7 (8)	5 (7)	
Position size, *N* (%)			
<50%	1 (1)	0 (0)	.001
50%–99%	53 (62)	24 (32)	
100%	17 (20)	27 (36)	
Data missing	15 (17)	24 (32)	

### 3.4 Ethics

The study was approved by the “Norwegian Centre of Research Data” (ref. No.: 56526). The study was conducted in accordance with ethical research guidelines. Respondents were provided with written information about the study's background and aims, and participation was voluntary. Delivering a completed survey form was taken as consent to participate in the study. Respondents were guaranteed anonymity, and all information was treated confidentially.

## 4 RESULTS

**FIGURE 1:**
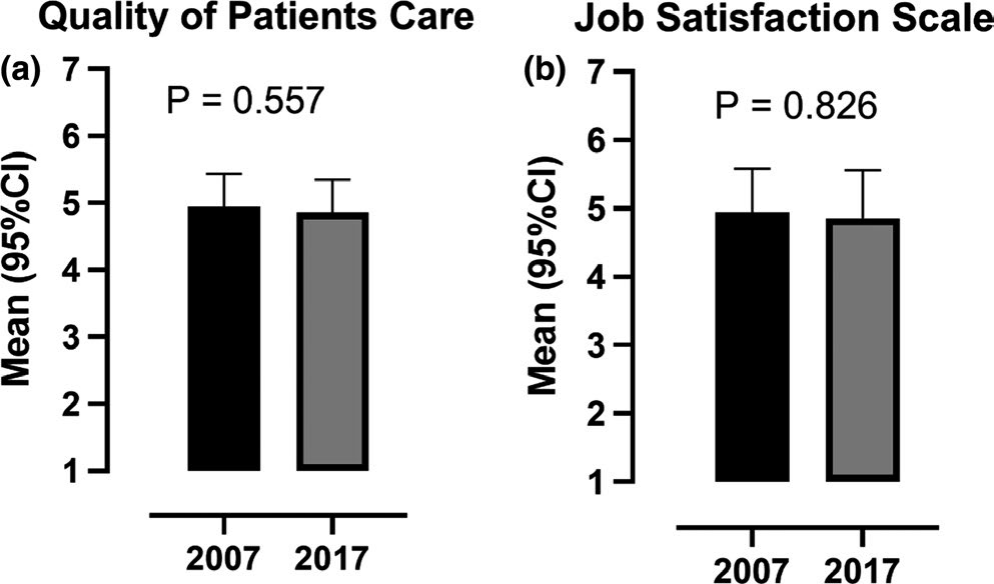
Quality of Patient Care and Job Satisfaction in 2007 and 2017

The participation rate in the study was 70.7% (*N* = 86) in 2007 and 52.8% (*N* = 75) in 2017. From 2007–2017, there were increases in nurses compared with auxiliary nurses, work experience and having a full‐time position, while there was no difference in the proportion of participants who had continuing education (Table 1). There were no differences in the QPC scores between 2007–2017 (Table 2 and Figure 1a). Furthermore, there were no differences in JSS scores between 2007–2017, except for the sub‐scale physical working conditions (Table 3 and Figure 1b).

**TABLE 2:** Crude mean (*SD*) of Quality of Patients Care (QPC) scores in 2007 and 2017, and adjusted differences between the two samples

Variables	2007 Mean, (*SD*)	2017 Mean, (*SD*)	Adjusted^a^ difference, 95% CI	*p*‐value
QPC total	5.4 (0.7)	5.2 (1.0)	−0.1 (−0.4, 0.2)	.557
Basic physiological needs	5.5 (1.2)	5.2 (1.3)	−0.3 (−0.7, 0.2)	.224
Communication and contact	4.9 (1.2)	4.7 (1.5)	−0.0 (−0.5, 0.4)	.910
Sleep, rest and peace	5.3 (1.0)	5.1 (1.3)	−0.0 (−0.4, 0.3)	.836
Infection control and hygiene	5.9 (0.8)	5.5 (1.2)	−0.2 (−0.6, 0.1)	.182
Correct treatment	5.8 (0.8)	5.7 (1.0)	−0.1 (−0.4, 0.3)	.740
Counselling and health promotion	5.4 (0.7)	5.2 (1.0)	0.1 (−0.3, 0.6)	.596

^a^ The following variables were adjusted in the analyses: ward, profession, work experience in the ward, continuing education and position size.

**TABLE 3:** Crude mean (*SD*) of Job Satisfaction Scale (JSS) scores in 2007 and 2017, and adjusted differences between the two samples

Variables	2007 Mean, (*SD*)	2017 Mean, (*SD*)	Adjusted^a^ mean difference, 95% CI	*p*‐value
JSS item	5.1 (0.9)	4.9 (0.9)	−0.0 (−0.4, 0.3)	.826
Amount of responsibility	5.2 (1.3)	5.0 (1.3)	0.0 (−0.4, 0.5)	.892
Variation of work	5.3 (1.2)	5.4 (1.3)	0.2 (−0.3, 0.6)	.419
Colleagues	5.9 (1.1)	5.6 (1.0)	−0.3 (−0.6, 0.1)	.164
Physical working conditions	4.8 (1.3)	4.3 (1.6)	−0.5 (−1.0, −0.0)	.043
Opportunity to use skills	5.3 (1.3)	5.1 (1.2)	0.0 (−0.4, 0.5)	.907
Overall job situation	5.6 (1.1)	5.6 (1.0)	0.1 (−0.3, 0.4)	.765
Freedom to choose own methods	5.1 (1.2)	4.7 (1.2)	−0.2 (−0.6, 0.2)	.379
Recognition of one's achievements	4.7 (1.5)	4.5 (1.4)	0.0 (−0.5, 0.5)	.938
Rate of pay	3.8 (1.6)	4.0 (1.6)	0.3 (−0.3, 0.8)	.310
Work hours	4.8 (1.3)	4.6 (1.3)	−0.1 (−0.6, 0.4)	.647

^a^ The following variables were adjusted in the analyses: ward, profession, work experience in the ward, continuing education and position size.

## 5 DISCUSSION

### 5.1 Changed organization—unchanged quality

It is an important finding that health professionals' ratings of the quality of care were good and comparable in 2007 and 2017. The Coordination Reform and other organizational changes that were undertaken in this period have resulted in more patients being treated for less time in the hospital. This may mean that the sickest patient group remains in hospital. Being able to handle more complex cases, thus, requires a higher level of expertise among healthcare personnel (Helsedirektoratet, 2012; Tyrholm et al., 2016). Based on this premise, one would expect that the competence of healthcare professionals has increased over this period to maintain the same quality of patient care.

A definite change was observed from 2007–2017 with respect to the staff's educational background. There was a significant increase in the number of nurses and a significant decrease in the number of auxiliary nurses by 2017. The hospitals in the study have not published data on the number of full‐time equivalent (FTEs) for nurses/auxiliary nurses in the organization, but, according to the HR Director, internal numbers from the payroll system show a substantial decline in FTEs for auxiliary nurses. This supports what was found in this study, and there is reason to believe that there has been a change in the competence of healthcare professionals, as there were more nurses and less auxiliary nurses in 2017 than in 2007.

If one assumes that there has been a change in professional competence, this may be related to a change in the needs of the organization after The Coordination Reform (Norwegian Ministry of Health & Care Services, 2008–2009) and other national guidelines (Norges forskningsråd, 2016). An organization needs to align and balance the need for different occupational groups (Førde, 2019). When more employees are nurses, it becomes easier to ensure adequate expertise according to the type of patient admitted (Andersen & Andersen, 2016).

Adequate nursing staff can increase quality of care, which is a factor in reducing patient mortality (Aiken et al., 2014). An increase in the number of nurses in the wards, as the study shows, therefore, may be a factor that helps maintain quality. No increase in hospital mortality has been observed since hospital reform was introduced in Norway (Norges forskningsråd, 2016).

If one argues, further, that enhancing competence is necessary to maintain nursing competence in a more complex workday with sicker patients, one would also expect an increase from 2007–2017 in the number of nurses with continuing education. Yet, this study found no increase in the percentage of staff who had completed further education, even though nursing tasks have become more specialized.

Complex care needs can reduce quality of care if one does not simultaneously raise competence (Sjetne et al., 2009). According to Tyrholm et al. (2016), nursing mangers say there is special need for advanced clinical competence in somatic departments to support good and sound patient care. The Hospital Trust has worked systematically and purposefully on quality work, and the management has focussed on improving competence through internal training and guidance about patient safety and work improvement (Vest, 2019). Thus, it appears that the Hospital Trust's internal training may have compensated for the lack of continuing education. It is also possible that the nursing staff has adapted to the changes. However, the question remains whether the quality of nursing could have increased if more nursing staff had completed continuing education.

Another factor that can help maintain the quality of care is staff experience (Wan et al., 2018). In this study, there were significantly more employees with more than ten years of experience in terms of both total years of experience and experience on specific wards in 2017 than in 2007. This means that there were more staff who had the expertise that could help them deal with complex issues and provide better patient care (Aiken et al., 2011). The clinical skills and knowledge of experienced nurses also make them role models for nurses with less experience (Kleiven et al., 2016; Wan et al., 2018).

This study found a significant change in employees in 100% FTE positions. In 2007, only 20.7% of all nurses worked in full‐time positions, whereas 36% held full‐time positions in 2017. When more nurses work full time, it means more continuity of work. Staffing is a way to increase patient safety and the quality of patient care (Førde, 2019).

### 5.2 Connection between quality of care and job satisfaction

The average scores for job satisfaction showed no significant change from 2007–2017. The 2017 data showed that many of the employees had worked in the same ward for a long time. This indicates there was increased stability in the workforce during the study period. A systematic review reported a significant connection between a stable labour force and job satisfaction (Li et al., 2018). Based on this finding, one may ask why job satisfaction had not increased, since the labour force had become more stable. It is possible that this lack of improvement in job satisfaction was due to organizational changes during this period. For example, The Coordination Reform may have led to more complex working conditions.

There was one domain of the JSS that changed significantly between 2007–2017—physical working conditions which showed a significant decrease in satisfaction from 2007–2017. This finding matches the findings of a national survey of job satisfaction and patient safety culture, where working conditions and physical work environment received the lowest score from staff (Vest, Nord, Midt‐Norge, & Sør‐Øst, 2018). If the physical environment on the ward does not change in accordance with the requirements of increased patient complexity, it can affect the staff's job satisfaction. This may be one reason why nursing staff were less satisfied with their physical working conditions in 2017 than in 2007.

Framework factors are related to how one feels about performing nursing duties (Kleiven et al., 2016; Li et al., 2018). Framework factors also include the physical working environment. For example, having 32% of the IKT equipment being older than 11 years (Førde, 2018), when it is recommended that no more than 10% of the IKT equipment should be older than 10 years (Jakobsen, Lind, Engebretsen, & Skogli, 2019). This is a problem because older equipment becomes less reliable and it becomes more difficult to adopt new technologies and solutions (Førde, 2018). Lack of a proper framework consumes resources (Jakobsen, Lind, Theie, & Skogli, 2018) and leads to a gap between what one needs and what one has available, which affects job satisfaction (Li et al., 2018).

Organizational changes can be experienced as challenging by both staff and management (Bernström, 2014). For example, must patient treatment be in line with the goals set by The Coordination Reform, which changed the distribution of tasks between specialist health services and primary health care (Norwegian Ministry of Health & Care Services, 2008–2009). This entailed, among other things, incorporating a new culture and new practice routines into the workplace (Bernström, 2014). Among other things, employees must work on adapting the patients and their relative's expectations when the total amount of beds in the health service is reduced. If there is no compliance between needs and expectations, this can lead to both ethical and practical dilemmas in everyday life. A sicker patient group that gets more advanced treatment in a specialist health service will, for example, need more screened treatment rooms, while this is not organized in the health enterprise, which has been partially protected and has older building stock. Even though there is a plan for single rooms, few facilities had instituted them at the time of the examination (Førde, 2017). This has increased the notion of shortcomings, but it takes time before measures are implemented. A larger part of nurse's work will be to prioritize between sick patients and organizing patient follow‐up. Achieving quality, therefore, involves the gap between professional ideal and practical everyday life (Orvik, 2015).

The nursing staff's job satisfaction has a major impact on how practitioners provide professional quality care in practical everyday life. Employees who are satisfied with how they do their work tend to emphasize a culture of patient safety (Rathert & May, 2007). Job satisfaction has a great impact on attitudes towards patients and thus on the quality of patient care (Bondevik, Hofoss, Husebø, & Deilkås, 2017; Elsous et al., 2016; Wang, Chou, & Lai, 2019). A nursing staff that is satisfied with its work is also more patient focused and makes fewer mistakes, with respect to medications, for example (Rathert & May, 2007).

### 5.3 Limitations and strengths

A weakness of the study is that the number of nursing staff who participated in the study was lower in 2017 than 2007. This may have different reasons. One could be a decline in participation rate in studies over time, something that is seen in population‐based studies (Jacobsen, Eggen, Mathiesen, Wilsgaard, & Njolstad, 2012; Krokstad et al., 2013). Another reason may be that workload forces the nursing staff to prioritize (Kleiven et al., 2016) and as a consequence answering questionnaires may be deprioritized. A consequence of the lower participant in 2017 is that sample selection could be skewed, which can affect the results (Amundsen, 2013).

Another weakness of the study is that it was not conducted at more frequent intervals. This could have provided valuable information about developmental trends. The groups (2007 versus 2017) are not the same persons over time, although a few of them might have participated in the study at each occasion.

Finally, although the repeated cross‐sectional design in this study display changes at the population level, assessing changes in the same individuals over time in a cohort study would also have been interesting. However, it is possible that the study's participation rate would have been lower with a non‐anonyms design and that outcomes in those who choose to stay at the wards would differ from those who left during the follow‐up period. Thus, there are strengths and limitation with both these designs.

The fact that the same method and measures were used at both points in time strengthens the reliability of the study. The study's findings from 2007 were consistent with concurrent findings from a larger sample (Sjetne et al., 2009). No study using similar measures has been conducted elsewhere in Norway after the introduction of The Coordination Reform, but one can assume that the hospitals of the Førde Hospital Trust are fairly representative of hospitals in Norway, so these findings can be generalized.

## 6 CONCLUSION AND IMPLICATION FOR PRACTICE AND RESEARCH

We found no difference in the quality of care or job satisfaction, from 5 years before–5 years after The Coordination Reform was introduced. The study's findings suggest that increased competence, greater work experience on the wards and higher FTE's may have compensated for increased pressure related to The Coordination Reform. Further research is needed to confirm these findings.

In our study, there was no increase in the percentage of staff who had completed further education, even though nursing tasks have become more specialized. One implication for practice may be that enhancing competence through specialized education is necessary to increase quality of care in a workday with more complex care needs. This study calls for further research to explore this assumption.

## CONFLICT OF INTEREST

None.

## AUTHOR CONTRIBUTIONS

IAaA, OTK, LK, MASP: Contributions to conception and design, or acquisition of data, or analysis and interpretation of data; drafting the manuscript or revising it critically for important intellectual content; final approval of the version submitted for publication. Each author has participated sufficiently in the work to take public responsibility for appropriate portions of the content; accountable for all aspects of the work in ensuring that questions related to the accuracy or integrity of any part of the work were appropriately investigated and resolved.
